# Supplementary material for: High-speed AFM height spectroscopy reveals µs-dynamics of unlabeled biomolecules
Source: Nat Commun. 2018 Nov 26;9:4983. doi: 10.1038/s41467-018-07512-3 (PMC6255864; doi:10.1038/s41467-018-07512-3)
Supplement: Supplementary file 1 — Supplementary Information [file 41467_2018_7512_MOESM1_ESM.pdf]

Supplementary Information for:

High-speed AFM height spectroscopy reveals µs-dynamics of unlabelled biomolecules

Heath *et. al.*

A5 Oligomer	$<d_p>$ *(nm)*	*w* (nm)
Monomer	9.4	10.2 ± 0.4
Dimer	13.3	14.2 ± 0.5
Trimer	16.2	17.3 ± 0.6
Tetramer	18.8	20.0 ± 0.6
Pentamer	21.0	22.3 ± 0.7

Supplementary Table 1. Average oligomer dimensions as determined from the molecular structures of A5 oligomers before ($<d_p>$) and after (*w*) convolution with a 1 ± 0.5nm AFM tip radius.

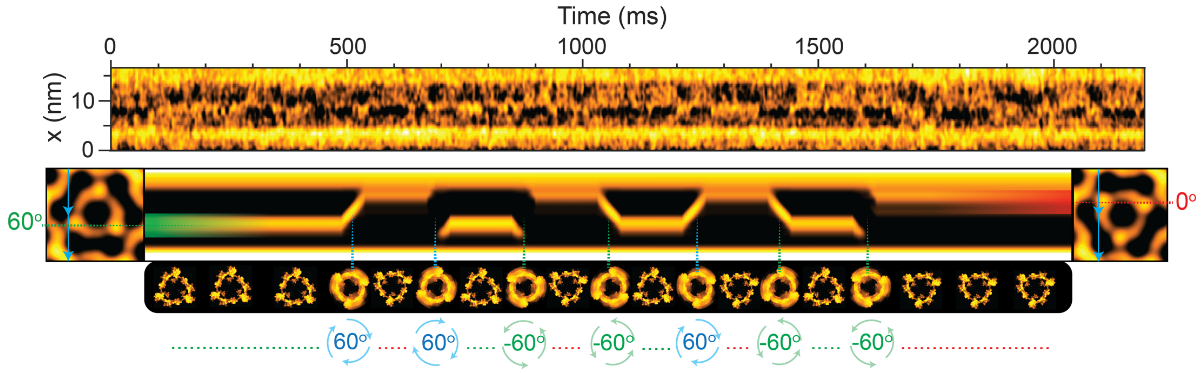

Supplementary Figure 1) Model Line scanning of the rotation of A5 at the 6-fold symmetry axis in the *p*6-lattice. Line scanning kymograph (top), and model line kymograph (middle). Model kymographs were created by rotating a structural model of the annexin lattice (after convolution to mimic a 1nm AFM tip radius) between the two preferred orientations (images labelled 60° and 0°). The model kymographs were created by plotting the profile indicated by the blue arrows over time during serval random 60° rotations with the trimer remaining stationary between rotations. Images and labels (bottom), 60 or -60°, indicate the positions where the model trimer undergoes either a clockwise or counter-clockwise rotation.

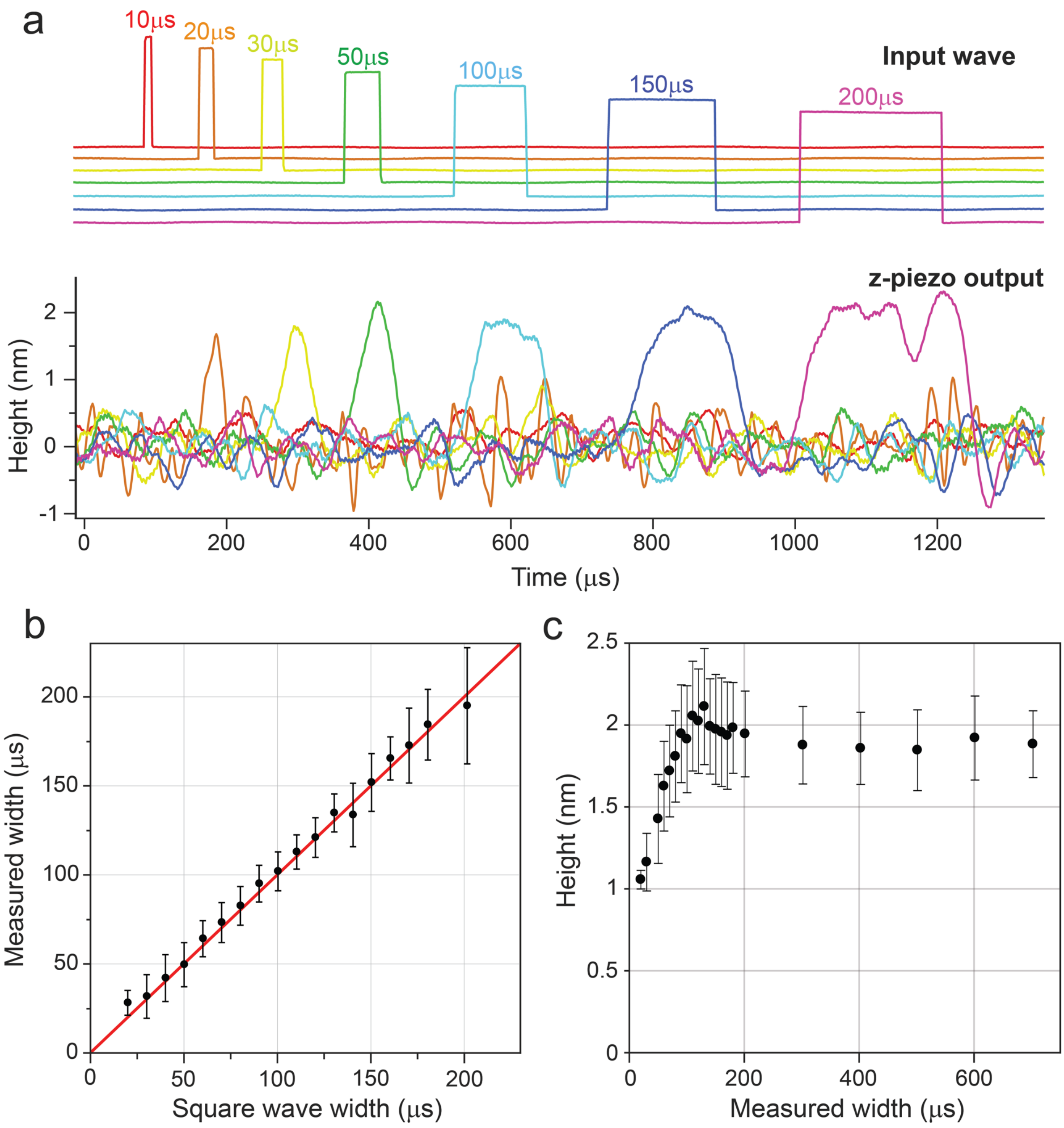

Supplementary Figure 2) z-feedback-loop response speed. a) Generated mock signals with varying square wave peak widths to simulate deflection caused by diffusion events under the tip (top) and the subsequent corresponding z-piezo response signals (bottom). For generated signals with widths <10μs no z-piezo response was observed. **b)** Measured z-piezo peak widths and **c)** average peak heights for varying mock signal peak widths. Data points are averages from n>30 measurements for each square wave width with error bars showing standard deviation (average error bar $\pm$ 12μs). For all measurements, the tip was held at a single x-y position on the surface of mica in experimental buffer conditions.

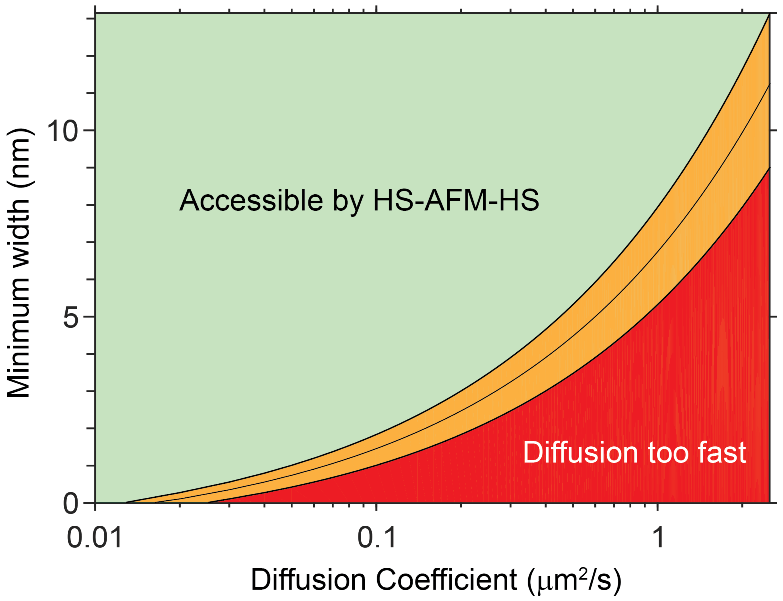

Supplementary Figure 3) Diffusion-dependent lateral molecule dimensions accessible by HS-AFM-HS. Red region classifies molecule sizes/diffusion rates which would diffuse under the tip with nominal dwell-times < 10μs and therefore would generally be undetectable. The green region classifies detectable molecules (plot assumes a AFM tip radius of 1nm).

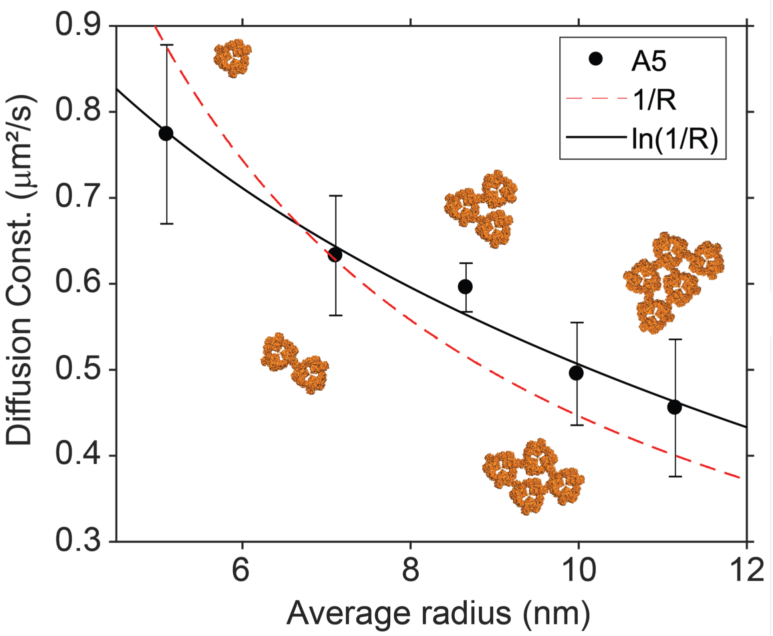

Supplementary Figure 4) Dependence of diffusion constant on A5 oligomers lateral radius. Diffusion constants were determined from the average peak positions in the dwell time distributions (mean +/- s.d.) using average A5 oligomer radii (average radii for various possible oligomer arrangements) for n = 1, 2…5 (A5, $A5_2$, $A5_3$, $A5_4$, $A5_5$). The solid line shows the fit of the data to the Saffman-Delbrück model (ln(1/R)). For comparison, the dashed line represents the fit to 1/R radial dependant diffusion.

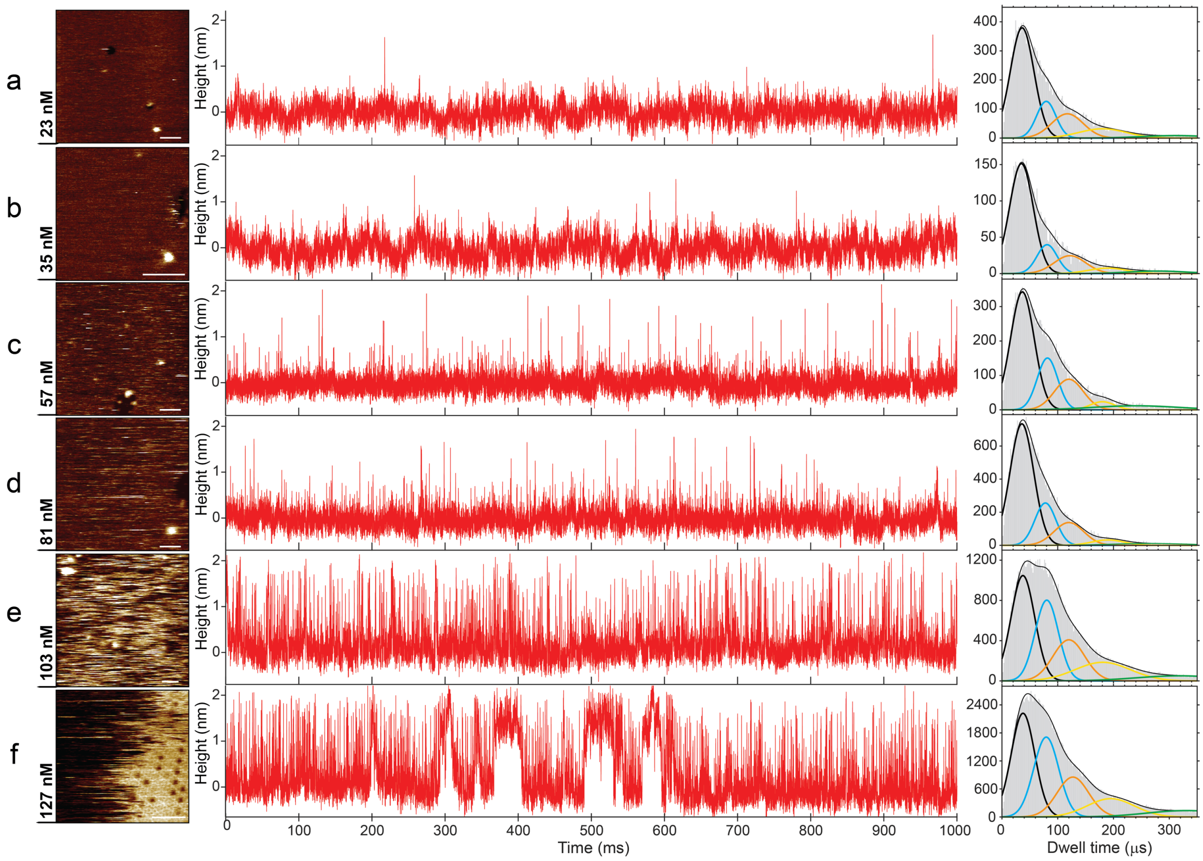

Supplementary Figure 5) HS-AFM-HS of self-assembly at model membranes for varying annexin-V concentrations. a, b, **c**, **d**, **e**, and **f)** HS-AFM image frames (left) and subsequently recorded height/time traces (middle) on the membrane (8:2 DOPC/DOPS) surface with 23nM (b), 35nM (c), 57nM (d), 81nM (e), 103nM (f) and 127nM (g) annexin-V in solution. Right: corresponding dwell time plots with residence time Gaussian fits from the diffusion of different sized molecules under the tip. All measurements were performed in the presence of 2mM $CaCl_2$. 1000ms height/time traces are example sections from longer, typically 60s, traces. Scale bars: 50nm. Full color scale: 4nm. Scale bars: 50nm.

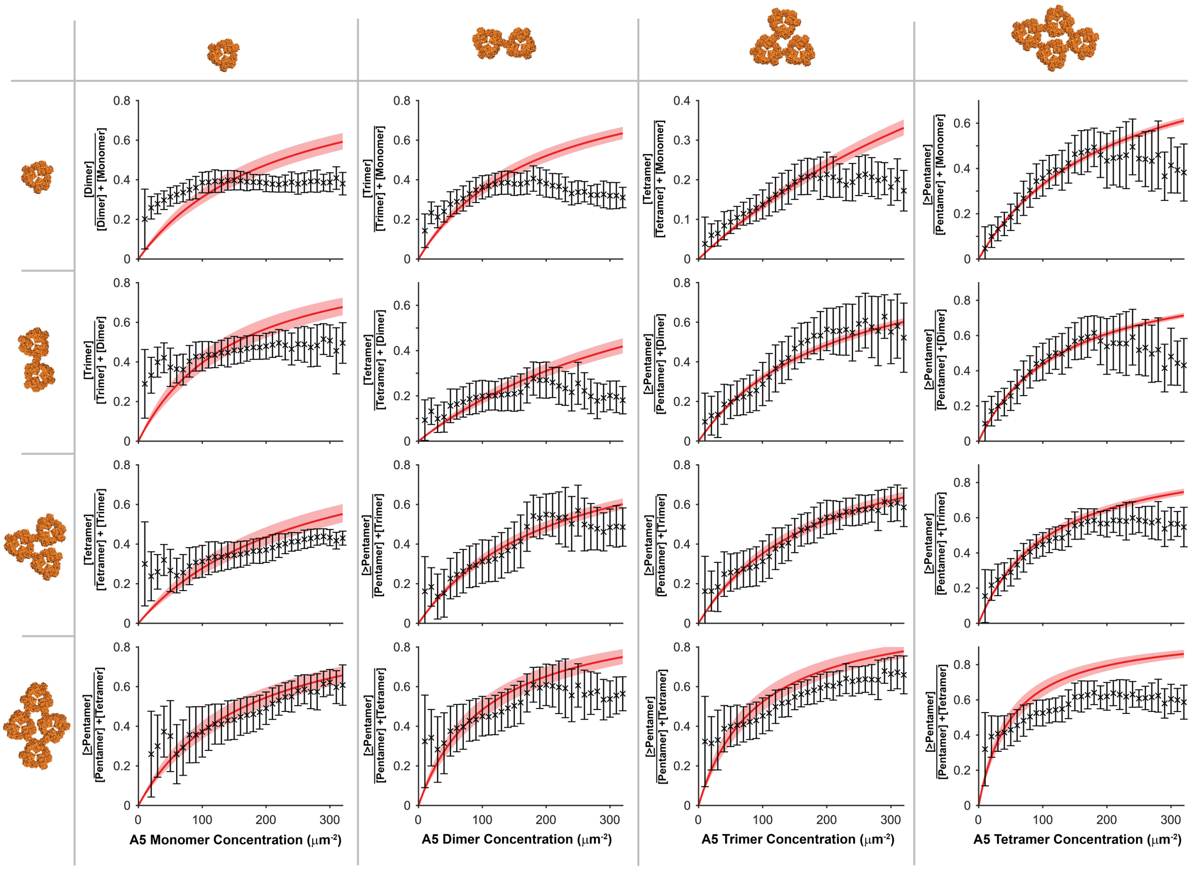

Supplementary Figure 6) 2D A5 binding curves for the determination of 2D dissociation constants K_d between A5 and higher order oligomers. Plots are arranged into a grid where the columns represent $[A]$ and rows represent the fraction of $[B]$ in complex, $\frac{[AB]}{[AB]+[B]}$. For example, the plot at row 1 column 2 corresponds to the formation of an annexin trimer-of-trimers $A5_3$ from the encounters of A5 and $A5_2$. Graphs are fitted with *eq. 5*. Data points are mean values +/- s.d. Fitting error is shown by 95% confidence interval fits displayed by the shaded red fill together with red line fit.

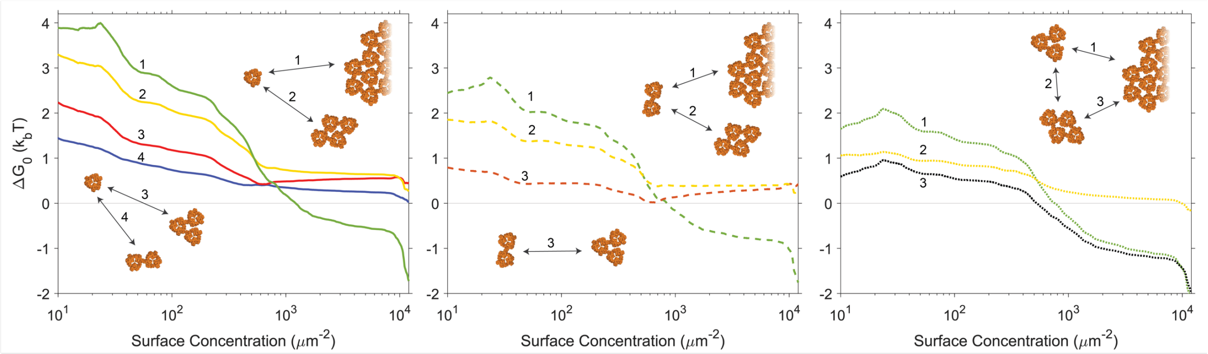

Supplementary Figure 7) Free energy differences between oligomer states as a function of surface concentration. The oligomer states are illustrated and labelled by numbers in each plot. Free energies are calculated using $\Delta G/K_bT = ln(c_n / c_m)$, where c_n and c_m stand for the concentration of the two oligomeric species that are compared.

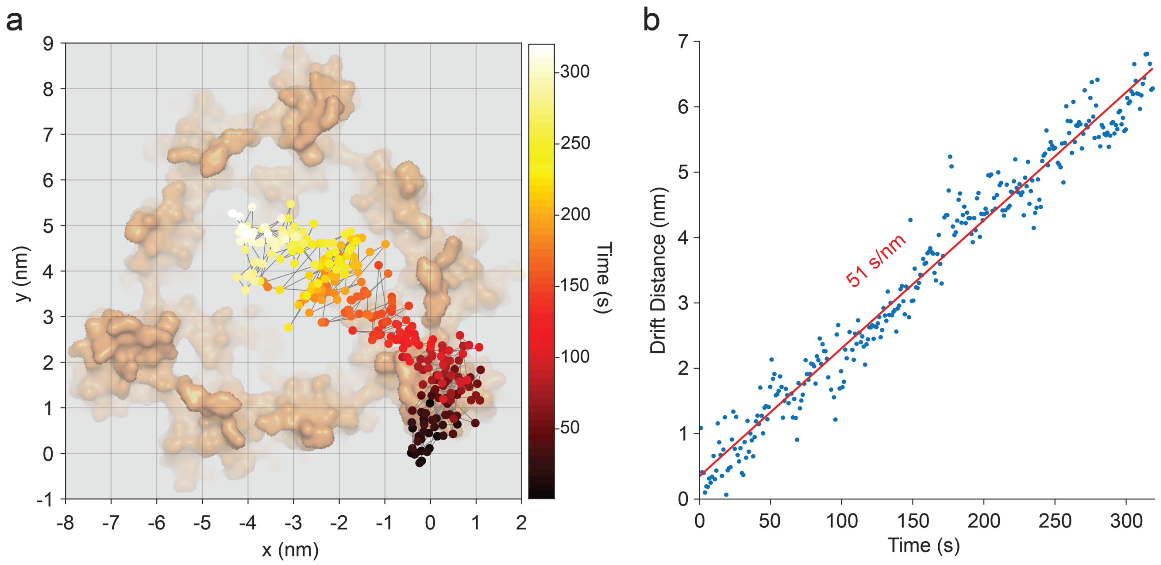

Supplementary Figure 8) Quantification of lateral drift. a) x-y position change during HS-AFM imaging as determined by image correlation alignment with subpixel interpolation of Supporting Movie 2. The translations are overlaid onto an annexin trimer (A5) to illustrate the scale of lateral drift during HS-AFM-HS measurements. **b)** Total drift distance from initial position over time with linear fit to determine a drift rate of 51s/nm (0.0195 nm/s). It should be noted that alignment was performed on images with 0.5nm/pix and thus apparent short timescale drift noise is expected to be due to alignment accuracy.
