# Supplementary material for: High-speed AFM height spectroscopy reveals µs-dynamics of unlabeled biomolecules
Source: Nat Commun. 2018 Nov 26;9:4983. doi: 10.1038/s41467-018-07512-3 (PMC6255864; doi:10.1038/s41467-018-07512-3)
Supplement: Supplementary file 3 — Description of Additional Supplementary Files [file 41467_2018_7512_MOESM3_ESM.pdf]

Description of Additional Supplementary Files

File Name: Supplementary Movie 1
Description: Visualization of the A5 non-p6-trimer rotation Description: High-resolution high-speed AFM image sequence of the rotation of individual A5 non-p6 trimers rotating within the p6-lattice interstices. Movie parameters: Image size: 120nm. Full color scale: 1nm. Image acquisition speed: 1s

File Name: Supplementary Movie 2
Description: Lateral drift of x-y position Description: 5min high-speed AFM image sequence the A5 lattice after image correlation to correct for drift. Movie parameters: frame size: 100nm. Full color scale: 1nm. Image acquisition speed: 1s
